# Supplementation with fibroblast growth factor 7 during *in vitro* maturation of porcine cumulus-oocyte complexes improves oocyte maturation and early embryonic development

**DOI:** 10.3389/fvets.2023.1250551

**Published:** 2023-11-07

**Authors:** Haomiao Zheng, Hyerin Choi, Dongjin Oh, Mirae Kim, Lian Cai, Ali Jawad, Sohee Kim, Joohyeong Lee, Sang-Hwan Hyun

**Affiliations:** ^1^Laboratory of Veterinary Embryology and Biotechnology (VETEMBIO), Veterinary Medical Center and College of Veterinary Medicine, Chungbuk National University, Cheongju, Republic of Korea; ^2^Institute of Stem Cell and Regenerative Medicine (ISCRM), Research Institute for Natural Science, Chungbuk National University, Cheongju, Republic of Korea; ^3^Department of Biological Sciences, College of Arts and Sciences, University at Buffalo, The State University of New York (SUNY), Buffalo, NY, United States; ^4^Graduate School of Veterinary Biosecurity and Protection, Chungbuk National University, Cheongju, Republic of Korea

**Keywords:** cytokine, cumulus-oocyte complexes, embryonic development, fibroblast growth factor 7, parthenogenetic activation, porcine embryos

## Abstract

*In vitro* generation of porcine embryos is an indispensable method in the realms of both agriculture and biomedicine. Nonetheless, the extant procedures encounter substantial obstacles pertaining to both the caliber and efficacy of the produced embryos, necessitating extensive research to *in vitro* maturation (IVM), the seminal commencement phase. One potentially fruitful approach may lie in refining the media and supplements composition utilized for oocyte maturation. Fibroblast growth factor-7 (FGF7), alternatively termed keratinocyte growth factor, is a theca-derived cytokine integral to folliculogenesis. This study aimed to examine the ramifications of supplementing FGF7 during the IVM phase. To determine the FGF7 location and its receptor in porcine ovaries, immunohistochemistry was executed based on follicle size categories (1–2, 3–6, and 7–9 mm). Regardless of follicle size, it was determined that FGF7 was expressed in theca and granulosa cells (GCs), whereas the FGF7 receptor was only expressed in the GCs of the larger follicles. During the IVM process, the maturation medium was supplied with various concentrations of FGF7, aiming to mature porcine cumulus-oocyte complexes (COCs). The data indicated a significant augmentation in the nuclear maturation rate only within the group treated with 10 ng/mL of FGF7 (*p* < 0.05). Post-IVM, the oocytes diameter exhibited a significant expansion in all groups that received FGF7 supplementation (*p* < 0.05). Additionally, all FGF7-supplemented groups exhibited a substantial elevation in intracellular glutathione levels, coupled with a noticeable reduction in reactive oxygen species levels (*p* < 0.05). With respect to gene expressions related to apoptosis, FGF7 treatment elicited a downregulation of pro-apoptotic genes and an upregulation of anti-apoptotic genes. The expression of genes associated with antioxidants underwent a significant enhancement (*p* < 0.05). In terms of the FGF7 signaling pathway-associated genes, there was a significant elevation in the mRNA expression of *ERK1, ERK2, c-*kit, and *KITLG* (*p* < 0.05). Remarkably, the group of 10 ng/mL of FGF7 demonstrated an appreciable uptick in the blastocyst formation rate during embryonic development post-parthenogenetic activation (*p* < 0.05). In conclusion, the FGF7 supplementation during IVM substantially augments the quality of matured oocytes and facilitates the subsequent development of parthenogenetically activated embryos. These results offer fresh perspectives on improved maturation and following *in vitro* evolution of porcine oocytes.

## 1. Introduction

The process of generating pigs in the lab, known as *in vitro*-production (IVP), holds great potential for various fields including biomedical, transgenic, and reproductive biotech research. Pigs, due to their genetic, anatomical, and physiological similarities to humans, are deemed as suitable donors for tissue and organs in areas such as xenotransplantation, regenerative medicine studies, as well as being models for human genetic diseases and biological factories for human proteins (1–3). The IVP method commonly employed to produce pig embryos involves three key stages: (1) *in vitro* maturation (IVM), (2) *in vitro* fertilization (IVF) or somatic cell nuclear transfer (SCNT), and (3) *in vitro* cultivation (IVC) of the embryos. Despite numerous techniques for creating pig embryos, the quality and potential for *in vitro* development remain subpar (4). Hence, further advancements are required to reliably produce high-quality pig embryos via IVP for biotech and biomedical research. IVM, being a foundational and non-negotiable step, its efficiency greatly influences the effectiveness of subsequent reproductive procedures (5–7). It is vital to create an environment mirroring *in vivo* conditions as closely as possible for IVM of oocytes, which is one of the factors affecting the effectiveness of IVP. These conditions are produced by diverse *in vitro* culture systems which include media with specific chemical constituents (5). Based on these findings, in-depth studies to improve the efficiency of IVM are necessary, and IVM research has focused on determining the optimal medium composition and various supplements to improve the oocyte maturation efficiency *in vitro*.

Cytokines are supplements that can increase the efficiency of oocyte maturation *in vitro* (8). Cytokines influence numerous physiological processes, including cell proliferation, differentiation, apoptosis, immune or hematopoietic responses, morphogenesis, angiogenesis, metabolism, and wound healing (9). Cytokines are essential regulators of ovarian physiology, especially during oocyte maturation (8). Specifically, cytokines assist in establishing an environment conducive to oocyte maturation. Each oocyte maturation stage involves a paracrine dialogue between the oocyte and granulosa/theca cells, primarily mediated by several hormones and cytokines (10, 11). Cytokines can be classified into various families or superfamilies based on their structural and functional characteristics.

Fibroblast growth factors (FGFs) are a family of cytokines who act through paracrine, autocrine, and endocrine pathways to promote multiple cellular responses, such as proliferation, growth, differentiation, and migration (12, 13). The FGF family comprises many members, including FGF2, FGF10, and FGF7, also known as keratinocyte growth factor (KGF). FGF receptor 2IIIb (FGFR2IIIb) is a unique FGF7 receptor (14, 15). Previous *in vitro* studies have shown that FGF7 stimulates the proliferation of several types of cells, such as keratinocytes (16), mammary epithelial (17), gastrointestinal epithelial (18), and bronchial (19). For reproduction, previous studies in sheep and cattle have revealed that FGF7 is expressed in theca cells, whereas FGFR2IIIb are expressed in the GCs of growing follicles (20, 21). Moreover, FGF7 promotes GCs proliferation in cattle and the survival, growth, and differentiation of cultured rat antral follicles (22, 23). In addition, FGF7 stimulates oocyte growth in bovine COC co-cultured with GCs (24).

The *in vitro* oocyte maturation system lacks membrane cells and their products, and the lack of these molecules may result in poor oocyte capacity in the *in vitro* production systems of bovine, humans, and possibly other mammals (25–27). Therefore, investigating the role of these molecules derived from membrane cells and oocytes is crucial. Moreover, the effects of FGF7 on the growth, maturation, and subsequent developmental potential of porcine oocytes have not yet been clarified. Therefore, this study aimed to investigate the effects of FGF7 on IVM, specifically on *in vitro* porcine oocyte nuclear maturation, intracellular glutathione (GSH) and reactive oxygen species (ROS) levels, and oocyte developmental capacity after parthenogenetic activation (PA).

## 2. Materials and methods

If not specifically mentioned, all chemicals and reagents used in this study were purchased from Sigma-Aldrich (St. Louis, MO, USA). About 200–300 porcine ovaries from the Dong-a food slaughterhouse were sampled at a time (Cheongju, Chungcheongbuk-do, South Korea). The study did not require approval from the Institutional Review Board of Chungbuk National University because the pig ovaries used were collected at Dong-a food slaughterhouse as a by-product. The ovaries were stored in thermally insulated containers filled with a 0.9% (w/v) NaCl solution to maintain an approximate temperature of 37°C. Post a brief journey to the lab, these ovaries were twice cleaned with the same solution. All materials used in the experiments were obtained from the same slaughterhouse.

### 2.1. Fluorescent immunohistochemical analysis of FGF7 and its receptors in porcine ovaries

A procedure involving fluorescent immunohistochemistry (IHC) was conducted as outlined before (28). The follicles were collected from selected ovaries and then classified according to their diameter into small (1–2 mm), medium (3–6 mm), and large (7–9 mm) were preserved in 10% formalin at 24°C for 48 h to prepare paraffin-embedded tissue slides. Tissue samples of 3 mm thickness were put in a cassette, washed three times with tap water. The preparation of paraffin blocks and section slides through dehydration and cutting was entrusted to the Laboratory Animal Center of Chungbuk National University. Paraffin section slides were chosen using microscope and then were preserved at 4°C until required.

The slides were dehydrated in a dry oven at 60°C for an hour before the paraffin removal. For deparaffinization, the slides were immersed in xylene twice for 5 min. The tissue sections, now free of paraffin, were washed with varying concentrations of ethanol (100, 90, 80, and 70%) for 5 min each for rehydration. After washing the rehydrated tissues under tap water for 10 min, they were heated in a preheated (100°C) sodium citrate buffer (10 mM, pH 6.0) for 20 min for antigen retrieval. After cooling at 24°C for 20 min, the slides were washed twice with Tris-buffered saline (TBS-T; pH 7.4) for 5 min. Prior to blocking, a hydrophobic boundary was drawn around the tissue using a hydrophobic pen (ImmEdgeTM Hydrophobic Blocking PAP Pen; Vector Laboratories, Inc., Burlingame, CA, USA). Slides were kept in a humidity-controlled chamber and blocking buffer (Cell Signaling Technology, Beverly, MA, USA) was applied to each slide and left at 24°C for 2 h. After washing the slides thrice with TBS-T for 5 min at 24°C, they were treated with a primary antibody and left overnight at 4°C in a humid chamber. The primary antibodies used were mouse anti-FGF-7 (sc-365440, diluted in blocking buffer; Santa Cruz Biotechnology, Santa Cruz, CA, USA) and mouse anti-Bek (C-8) (sc-6930, diluted in blocking buffer; Santa Cruz Biotechnology).

The next day, the slides were washed thrice for 5 min with TBS-T to remove the primary antibody. Then a secondary antibody solution was added and incubated at 24°C for an hour. Goat anti-mouse IgG (H + L) and Alexa FluorTM 488 (A11029; Invitrogen, Carlsbad, CA, USA) were the secondary antibodies used. The slides were then washed thrice for 5 min at 24°C with TBS-T, and Hoechst 33342 was used to counterstain the cell nuclei. Finally, an anti-fade mounting solution (Molecular Probes, Inc., Eugene, OR, USA), was used to mount each slide. The ovarian tissue sections were then viewed with a confocal laser microscope (Carl Zeiss, Thornwood, NY, USA), and all the images were examined using the ZEN (blue edition; Version 3.7) software program.

### 2.2. Oocyte collection and *in vitro* maturation of porcine oocytes

The pig ovaries were gathered, cleaned as outlined in materials section, and then rinsed twice with a 0.9% (w/v) NaCl solution. Ovaries from the same batch that had not been selected for IHC were used for this study. Cumulus-oocyte complexes (COCs) and follicular fluid from pigs were aspirated from follicles 3–6 mm deep and put into a 15 mL test tube kept at 37°C. After settling for 5 min at this temperature, the deposit was mixed again in a Tyrode medium buffered with HEPES and containing 0.05% polyvinyl alcohol (TLH-PVA) and was rinsed twice more. A stereomicroscope aided in selecting the COCs, with only those having at least three layers of densely packed cumulus-coated oocytes chosen. Around 60 of these selected COCs were put into four-well culture dishes (Nunc, Roskilde, Denmark), each containing 500 μL of maturation medium. The maturation medium included TCM199 medium (Invitrogen, Carlsbad, CA, USA), cysteine, sodium pyruvate, epidermal growth factor, kanamycin, insulin, equine chronic gonadotropin, human chorionic gonadotropin (hCG) (Intervet, Boxmeer, Netherlands), and polyvinyl alcohol. During IVM, the chosen COCs were cultured in a humid incubator at 39°C, with 5% CO_2_, and 95% air. The COCs were cultured for about 42 h, with the first 22 h in a medium containing eGG and hCG and the following 20 h without any hormones. FGF7 was added to the maturation medium at concentrations of 0-, 1-, 10-, and 100 ng/mL throughout the maturation period. After this period, the cumulus cells (CCs) were separated from the oocytes using 0.1% hyaluronidase in the IVM medium, and the oocytes were rinsed with TLH-PVA. Oocytes that had released the first polar body were classified as mature.

### 2.3. Evaluation of oocyte diameter

The size of pig oocytes was determined using a method previously documented (29). Briefly, after IVM, photographs of each set of denuded oocytes were captured at a 200 × magnification using a digital camera (DS-5Mc-L1; Nikon, Tokyo, Japan) attached to an inverted microscope (TE-300; Nikon). The dimensions of each part of the matured oocytes were assessed using ImageJ (ImageJ, Version 1.53t; National Institutes of Health, Bethesda, MD, USA). The oocytes size was then calculated as follows:


$$\begin{array}{l} Porcineoocytediameter=\left(A+B\right)/2. \end{array}$$

Each oocyte was measured twice (A, B), and the mean value of these two measurements was utilized, with the longest circumference being considered for both measurements, and the intersect of the two measurements forming a right angle.

### 2.4. Evaluation of nuclear maturation of pig oocytes

The nuclear maturation rate was assessed after 40–42 h of IVM, which was supplemented with FGF7. The cumulus cells were detached from the oocytes by adding 0.1% hyaluronidase to the IVM medium and rinsing with TLH/PVA. Every group of oocytes, denuded oocytes were then gathered, stained with TLH/PVA containing 10 μg/mL of Hoechst-33342 for 10 min, and inspected using a Nikon fluorescence microscope (Tokyo, Japan). Oocytes were categorized into germinal vesicles (GV), metaphase I (MI), anaphase/telophase I (A/TI), and metaphase II (MII) to evaluate meiotic maturation. Oocytes which had completed the expulsion of the first polar body were regarded as mature. This experiment was conducted three times for validation.

### 2.5. Evaluation of intracellular GSH and ROS levels

Mature oocytes were gathered 42–44 h post IVM, and their internal levels of glutathione (GSH) and reactive oxygen species (ROS) were evaluated using a previously described method (30). Cell Tracker Blue 4-chloromethyl-6, 8-difluoro-7-hydroxycoumarin (CMF2HC) and the 2', 7'-dichlorodihydrofluorescein diacetate (H2DCFDA) (Invitrogen, Carlsbad, CA, USA) indicators were used to detect the GSH and ROS levels within the ooplasm, respectively. Twenty oocytes were placed in TLH/PVA containing 10 μM of CMF2HC dye, then incubated in darkness for 30 min. After being washed thrice with TLH/PVA, the oocytes were placed in a 10 μL drop of TLH/PVA and their levels were evaluated using an epifluorescence microscope (TE300; Nikon) with an ultraviolet filter 370 nm for GSH. ROS was measured using the same method as for GSH by H2DCFDA dye, and the ROS level was detected using an epifluorescence microscope (TE300; Nikon) with ultraviolet filter 460 nm. The fluorescence intensity was measured and adjusted to match that of control oocytes using ImageJ software (Version 1.53t; National Institutes of Health). This process was conducted in three separate trials.

### 2.6. Evaluation of FGF7 containing media on CCs and oocytes mRNA expression post IVM using qRT-PCR

CCs and oocytes were harvested after 40–42 h of IVM in a medium containing FGF7 to study the shift in mRNA expression post-IVM. The CCs and oocytes were gently separated from the 60 COCs and from each other using soft pipetting with 0.1% hyaluronidase. Before analysis, all cells were twice rinsed in DPBS, transferred to 1.5 mL microfuge tubes, and then stored at −80°C. Quantitative polymerase chain reaction (qPCR) was carried out to assess the expression of specific genes related to diverse functions, including apoptosis (*BAX* and *BCL2L1*), antioxidant activity (*NRF2, NQO1, HMOX1*, and *GCLC)*, cumulus expansion or proliferation (*PTX3, CD44, Has2, PCNA*, and *Cx43*), FGF7 signaling pathway (*ERK1, ERK2, PIK3R1, AKT1, c-kit*, and *KITLG*), and oocyte secreted factors (*GDF9* and *BMP15*). Supplementary Table 1 lists all primer sequences.

RNA was extracted using TRIzol reagent (TaKaRa Bio, Otsu, Shiga, Japan), then converted to complementary DNA (cDNA) using a reverse transcription master mix (Elpis Bio, Daejeon, Korea) as the manufacturer's instructions. The qPCR mixture for the CFX96 Touch RealTime PCR Detection System (Bio-Rad, Hercules, CA, USA) contained 1 μg of synthesized cDNA, 2 × SYBR Premix Ex Taq, and 10 pM of specific primers (Macrogen, Seoul, South Korea). The reactions were executed for 40 cycles at varying temperatures: 95°C for 15 s for denaturation, 59°C for 15 s for annealing, and 72°C for 24 s for extension. Relative quantification was performed at a constant fluorescence intensity using threshold cycle-based techniques. The relative mRNA expression (R) was calculated using this formula: R = 2^−[Δ*Ctsample* − Δ*Ctcontrol*]^. The R values for each gene were standardized to the R values for *GAPDH* in CCs and *RN18S* in oocytes. A minimum of three repetitions were carried out for statistical analysis.

### 2.7. Parthenogenetic activation of porcine oocytes

After IVM, Metaphase II (MII) oocytes from each group were chosen and rinsed with a calcium-free TLH/PVA medium. The process of Parthenogenetic activation (PA) was carried out using a standard method (30). MII oocytes were rinsed twice in a 280 mM mannitol solution containing 0.01 mM CaCl_2_ and 0.05 mM MgCl_2_. Between the chamber electrodes the MII oocytes were placed in 2 mL of a 260 mM mannitol solution containing 0.01 mM CaCl_2_ and 0.05 mM MgCl_2_. The chamber was connected to an electrical pulse generator (LF101; Nepa Gene, Chiba, Japan), and the MII oocytes were stimulated by two direct-current pulses of 60 s at 120 V/mm each. Following PA, activated oocytes from all groups were immediately transferred to an *in vitro* culture (IVC) medium (30), composed of porcine zygote medium containing 5 μg/mL cytochalasin B. The oocytes were incubated for 4 h at 39°C in a humid atmosphere composed of 5% CO_2_ and 95% N_2_. The oocytes were then rinsed twice in a fresh IVC medium and placed in 25 μL of fresh IVC medium, with 10 oocytes per droplet, and covered with mineral oil. After 48 h (day 2) and 96 h (day 4), the IVC culture medium was replaced with 10% fetal bovine serum. Embryos were cultivated for a total of 7 days at 39°C in a humid incubator consisting of 5% O_2_, 5% CO_2_, and 90% N_2_. This experiment was performed thrice.

### 2.8. Developmental competence evaluation and total blastocyst cell count

On the second day, the division of the Parthenogenetic activation (PA) embryos was assessed. Developing embryos were divided into 2–3, 4–5 and 6–8 cell embryonic stages, one-cell embryos, and fragmented embryos. On the seventh day, the formation of blastocysts was evaluated (30). Based on their development, the blastocysts were divided into early blastocyst, expanded blastocyst, and hatched blastocyst stages. To count the total number of cells, the blastocysts were rinsed with TLH/PVA, fixed in 4% paraformaldehyde in PBS/PVA, and stained for 10 min with 5 g/mL Hoechst-33342 on the seventh day. The blastocysts were then fixed in an anti-fade mounting solution (Molecular Probes, Inc., Eugene, OR, USA), placed on glass slides, and captured with a digital camera (DS-5Mc-L1; Nikon, Tokyo, Japan) connected to a fluorescence microscope (TE-300; Nikon) equipped with a UV filter of 370 nm. The counting of the blastocysts was conducted using the ImageJ software (Version 1.53t; National Institutes of Health, Bethesda, MD, USA). The procedure was replicated thrice.

### 2.9. Statistical analysis

Unless otherwise specified, all experiments were performed at least thrice. Data were analyzed statistically using SPSS version 12.0 (SPSS, IBM, Armonk, NY, USA). GraphPad Prism (GraphPad Software, San Diego, CA, USA) was used to plot data. After a one-way ANOVA, the data were examined using Duncan's multiple-range test. Data are expressed as the mean ± standard. Differences were considered statistically significant at *p* < 0.05.

## 3. Results

### 3.1. Localization of FGF7 and its receptor in porcine ovarian follicular cells

**Figure 1:**
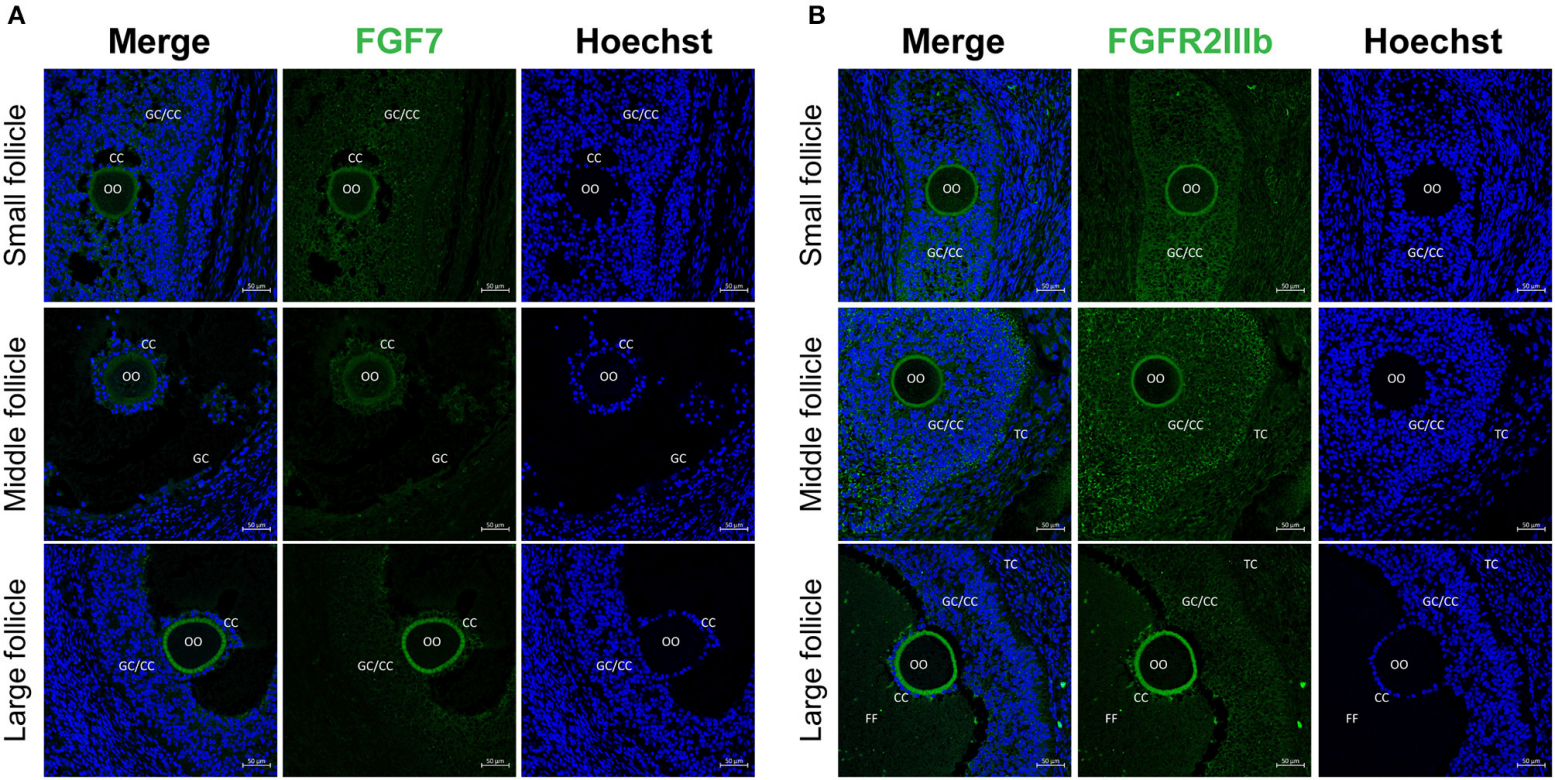
Localization of FGF7 and its receptors in porcine ovaries via fluorescence immunohistochemistry. **(A, B)** Identification and localization of FGF7 and its receptors in porcine ovaries via fluorescence immunohistochemistry. Pig ovarian tissues were divided into small (1–2 mm), medium (3–6 mm), and large (7–9 mm) sections according to follicle size. **(A)** FGF7. **(B)** FGFR2b. TC, theca cells; CCs, cumulus cells; GCs, granulosa cells; FF, follicular fluid.

Our results revealed that, FGF7 was expressed in the GCs, CCs, and oocytes of small and medium follicles. In large follicle sections, FGF7 was strongly expressed in the zona pellucida of oocytes but weakly expressed in CCs and GCs (Figure 1A). For the FGF7 receptor, a stable expression was observed in the GCs, CCs, and oocyte zona pellucida from small to large follicle sections (Figure 1B). Therefore, FGF7 and its receptor were exclusively expressed in the cytoplasm of CCs and GCs.

### 3.2. Effect of FGF7 supplementation on oocyte nuclear maturation during IVM

**Figure 2:**
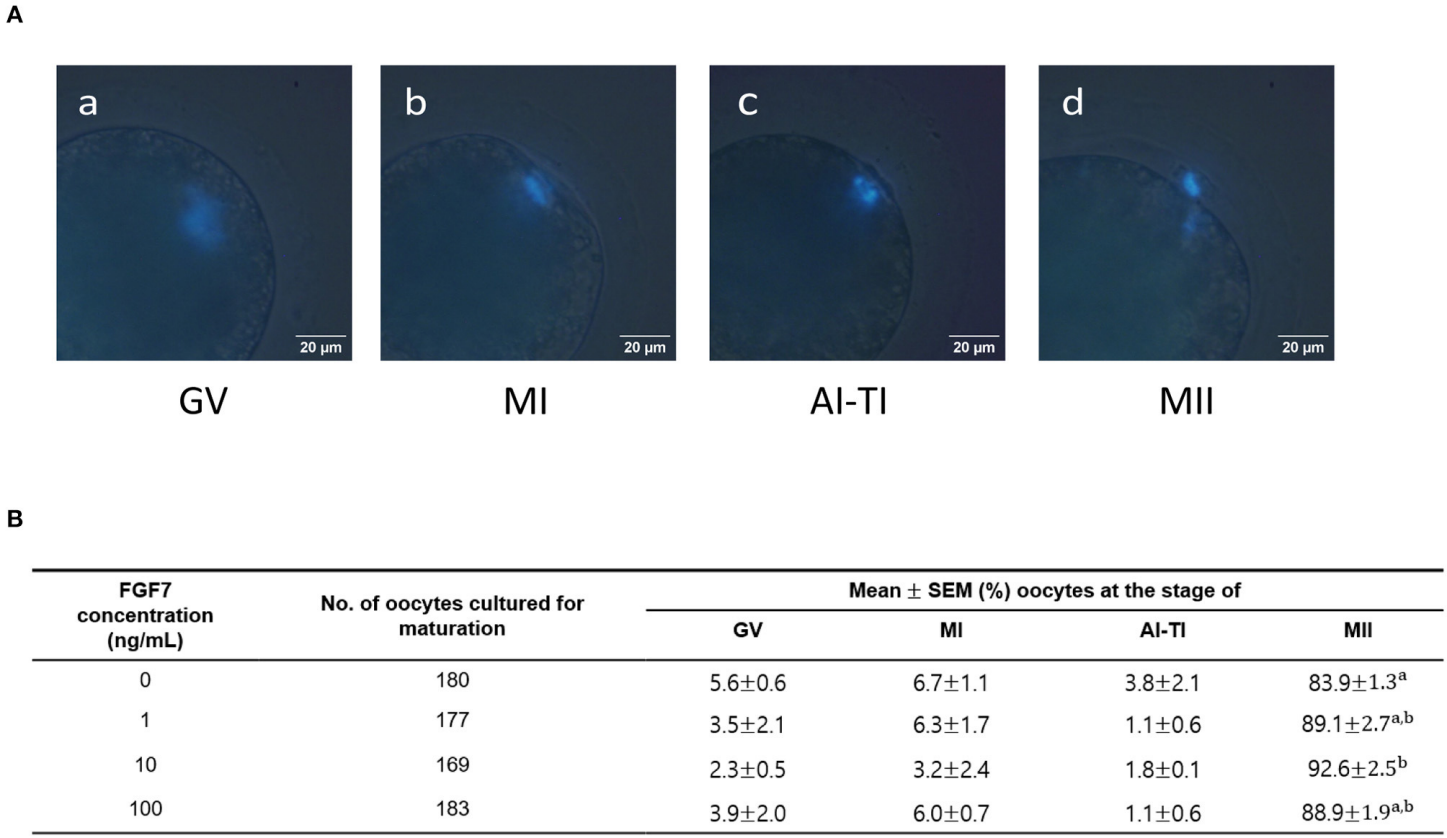
Effect of FGF7 supplementation in maturation medium on nuclear maturation. **(A)** Different nuclear maturation stages of oocytes. (a) Germinal vesicle (GV); (b) metaphase I (MI); (c) anaphase I and telophase I (AI-TI); (d) metaphase II (MII). **(B)** Summary of nuclear maturation stages after IVM. The nuclear maturation stages of oocytes were measured after 42 h, replicated thrice. ^a, b^Values with different superscripts within a column differ significantly (*p* < 0.05).

Four mature oocyte stages were evaluated to determine the effect of FGF7 treatment on the nuclear maturation of porcine oocytes during IVM: GV, MI, A/TI, and MII. Figure 2A shows an example of the classification of each stage of oocyte nuclear maturation used. The group supplemented with 10 ng/mL FGF7 had a significantly higher MII rate than the control group (92.6 ± 2.5%; *p* < 0.05). In contrast, no statistically significant differences between the other FGF7-treated groups and the control group were observed (Figure 2B).

### 3.3. Effect of FGF7 supplementation on oocyte diameter during IVM

**Figure 3:**
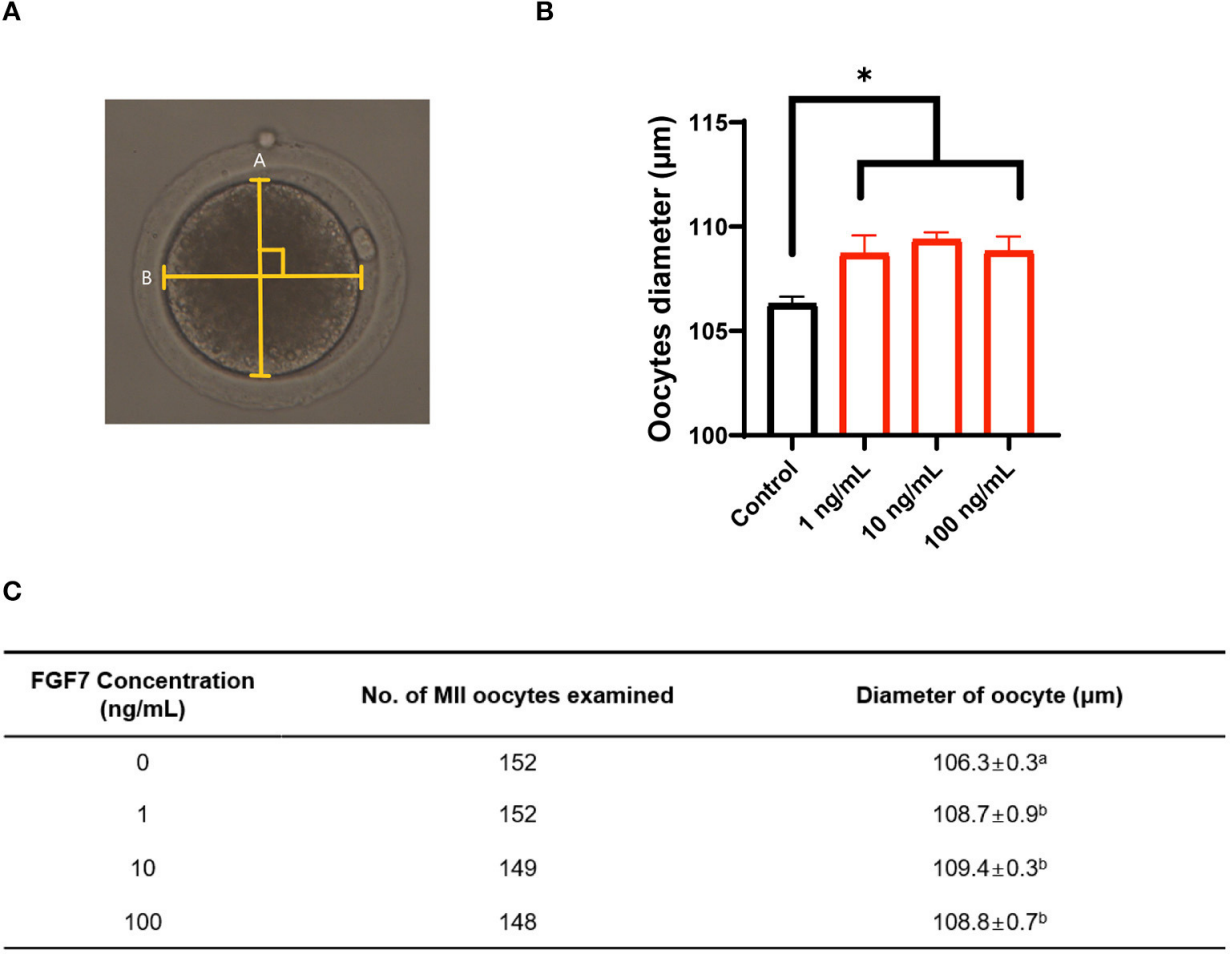
Effect of FGF7 supplementation during *in vitro* maturation (IVM) on size of porcine oocytes. **(A)** Schematic diagram of the measurement of porcine oocyte diameter after IVM. The oocyte was measured twice, and the average of the measurements was used. The longest circumference was used, and the crossover of the measurements was 90°. **(B)** Quantified diameter of mature porcine oocytes after 42 h of IVM supplemented with various concentrations of FGF7. **(C)** Evolution of oocyte diameter under different concentrations of FGF7 treatment and the number of measured oocytes. For all graphs, the value represents the mean ± SEM. Asterisks indicate statistical significance (*p < 0.05). ^a, b^Values in the same column with different superscript letters are significantly different (*p* < 0.05). Statistical significance was determined using one-way ANOVA. Experiments were repeated thrice.

The diameter of MII oocytes were measured according to the schematic in Figure 3A to determine the effect of different concentrations of FGF7 on oocyte maturation during IVM. On studding the effect of FGF7 supplemented with different concentrations to the culture media during IVM of porcine oocyte, we find that the diameters of all FGF7-supplemented oocytes were larger than those of untreated oocytes (*p* < 0.05; Figures 3B, C).

### 3.4. Effect of FGF7 supplementation during IVM on intracellular GSH and ROS levels

**Figure 4:**
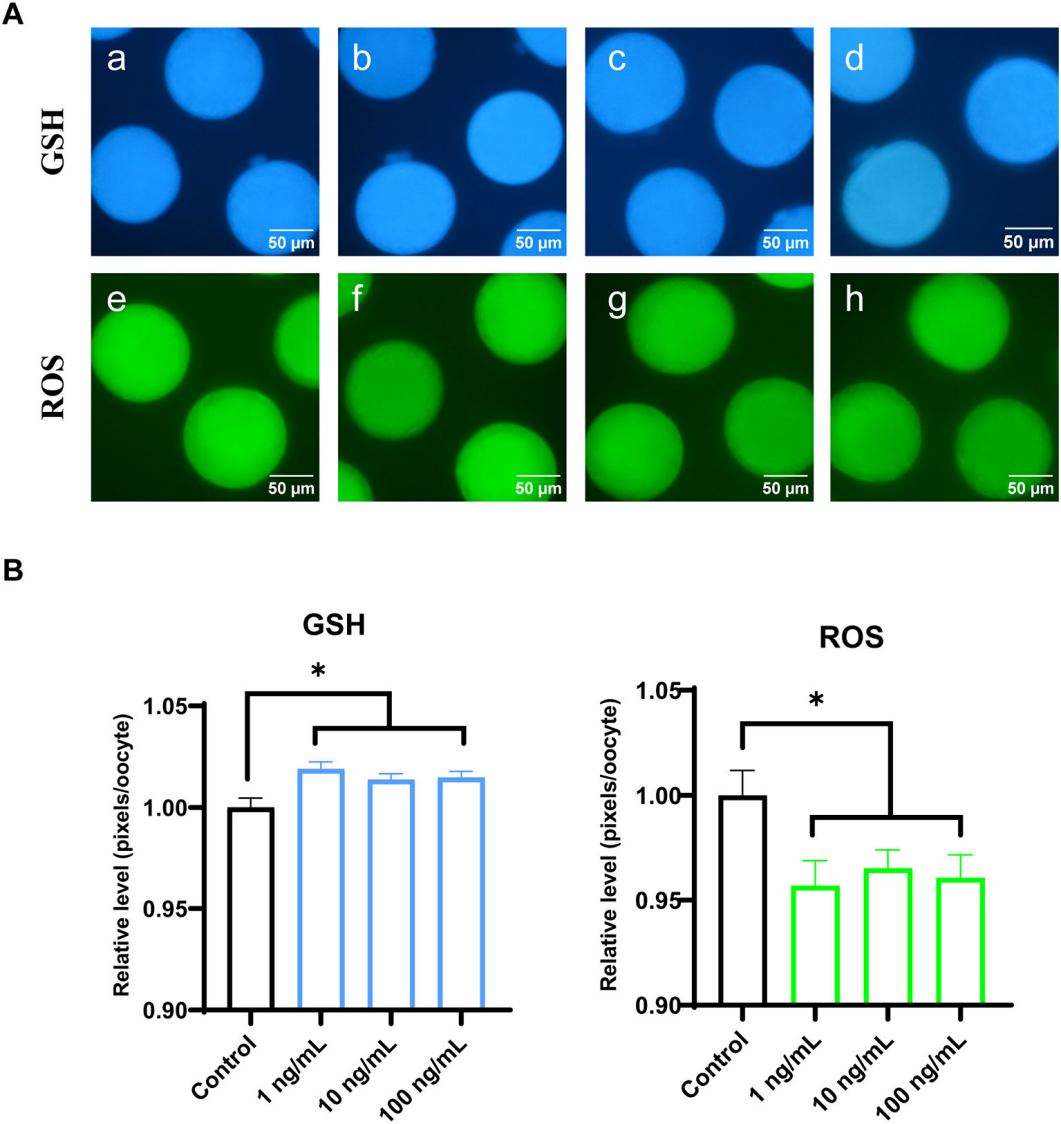
Epifluorescent photomicrographic images of *in vitro* mature porcine oocytes. **(A)** Oocytes were stained with Cell Tracker Blue to detect intracellular GSH levels (a–d) and stained with 2′, 7′-dichlorodihydrofluorescein diacetate to detect ROS (e–h). Metaphase II oocytes derived from maturation medium supplemented with FGF7 (0, 1, 10, and 100 ng/mL). **(B)** The relative levels of intracellular GSH and ROS in *in vitro* mature porcine oocytes among the FGF7 treated groups. Asterisks indicate statistical significance (*p < 0.05). The experiments were performed in triplicate. GSH samples, *N* = 60; ROS samples, *N* = 60.

To evaluate cytoplasmic maturation of porcine oocytes, intracellular GSH and ROS levels were measured in MII oocytes from the maturation medium supplemented with varying concentrations of FGF7 following IVM. Results of Figure 4A shows that, all FGF7-treated groups had significantly higher intracellular GSH levels than the control group (*p* < 0.05). Compared to the control group, intracellular ROS levels were notably decreased (*p* < 0.05) in all the FGF7-treated groups (Figure 4B). Thus, FGF7 supplementation during IVM remarkably altered the intracellular GSH and ROS levels in mature oocytes.

### 3.5. Effect of FGF7 supplementation on gene expression levels in CCs and oocytes during IVM

**Figure 5:**
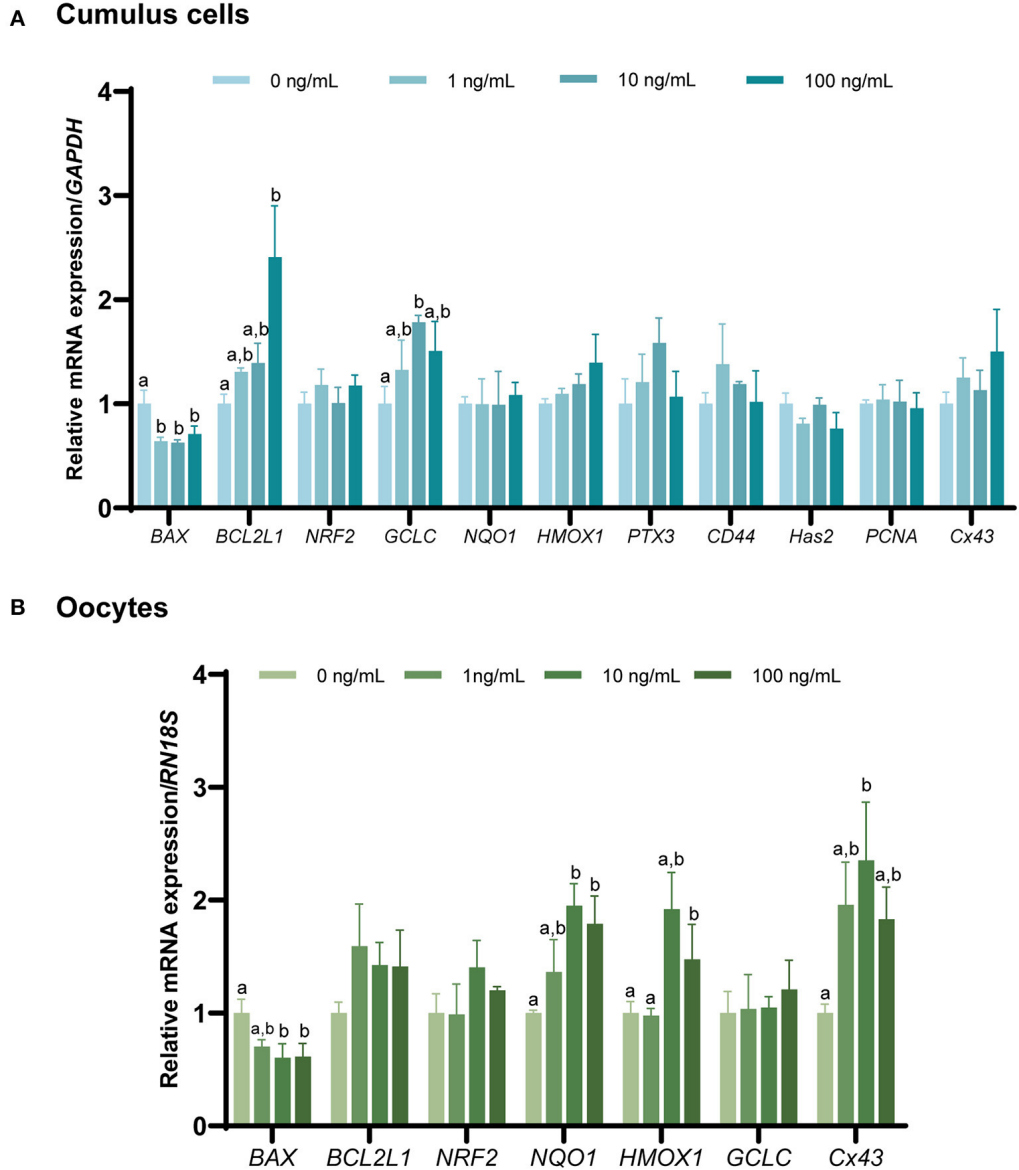
The mRNA expression levels (mean ± SEM) of apoptosis-, antioxidant-, cumulus expansion, and proliferation-related genes. *BAX, BCL2L1, NRF2, NQO1, HMOX1, GCLC, PTX3, CD44, Has2*, and *PCNA* expression levels were assessed in cumulus cells **(A)**, and of *BAX, BCL2L1, NRF2, NQO1, HMOX1*, and *GCLC* expression levels assessed in oocytes. **(B)** Supplemented with various concentrations of FGF7 after IVM. The experiment was performed in triplicate. Bars with different letters (^a, b^) indicate significant differences (*p* < 0.05).

Changes in gene expression in the CCs and oocytes were analyzed to determine the effect of FGF7 supplementation on porcine oocytes and CCs during IVM (Figures 5A, B). *BAX* mRNA expression was significantly decreased in all FGF7-treated CCs and oocytes (*p* < 0.05). In addition, the expression of the anti-apoptosis-related gene *BCL2L1* increased substantially (*p* < 0.05) in CCs treated with 100 ng/mL FGF7. In contrast, the *BCL2L1* levels in all oocyte treatment groups were not significantly different from those in the control group (*p* > 0.05). Among the antioxidant-related genes in the CCs, only the *GCLC* level was significantly increased in the 10 ng/mL group compared to that in the control group (*p* < 0.05). However, the other antioxidant-related genes did not change significantly. In oocytes, the 10 and 100 ng/mL treatment groups exhibited a significant increase in the *NQO1* expression level compared to those in the control group (*p* < 0.05). Additionally, the 100 ng/mL treatment group exhibited a significant increase in *HMOX1* levels (*p* < 0.05). The remaining antioxidant-related genes in the oocytes exhibited no significant changes. Within the genes associated with CC expansion and proliferation, all FGF7 treatment groups in CCs showed no significant differences in *PTX3, CD44, Has2, PCNA*, and *Cx43* (*p* > 0.05). In oocytes, *Cx43* was significantly increased in the 10 ng/mL FGF7-treated group (*p* < 0.05, Figures 5A, B).

**Figure 6:**
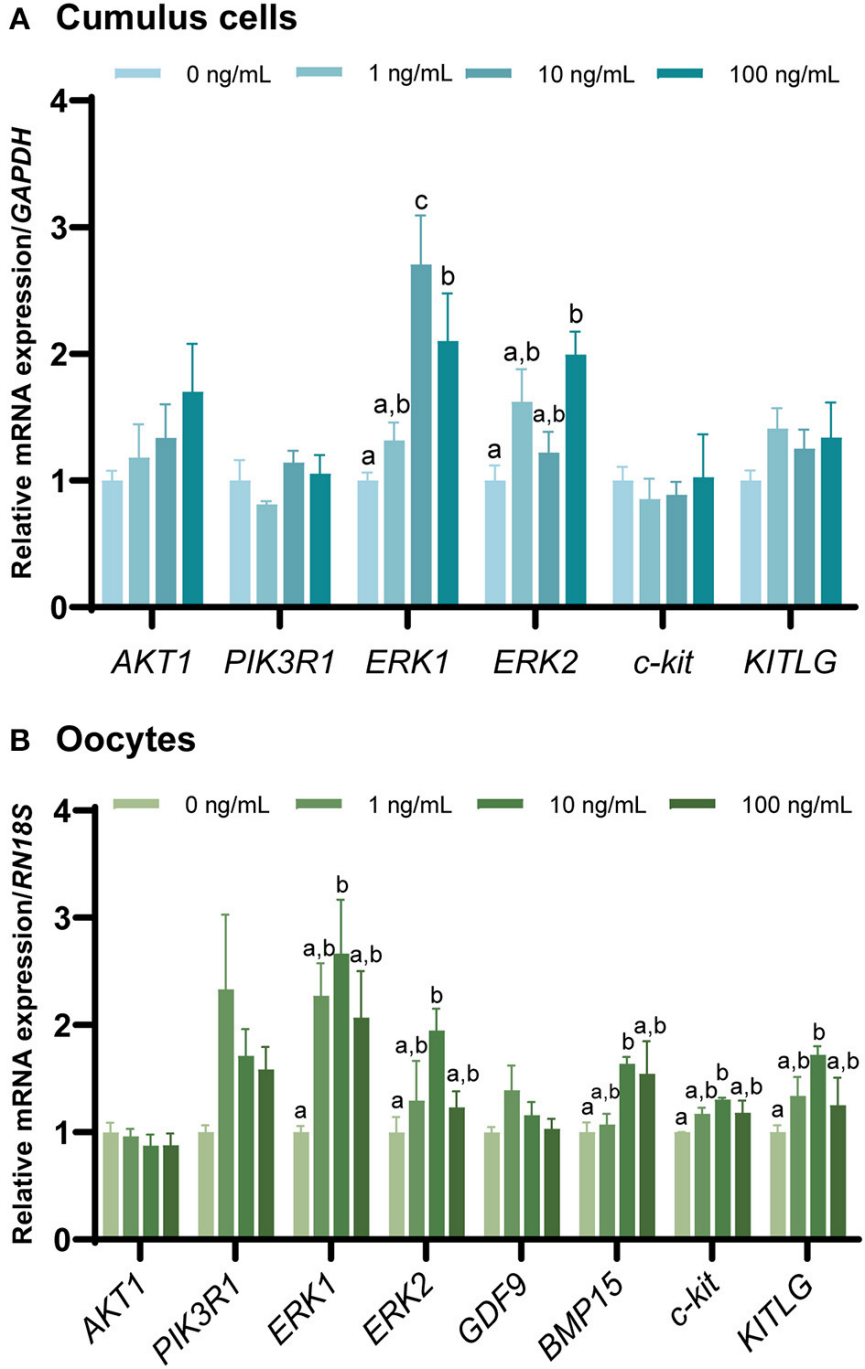
The mRNA levels of FGF7 signaling pathway-related genes and oocyte-secreted factors. mRNA levels of *AKT1, PIK3R1, ERK1, ERK2, c-kit, KITLG, GDF9*, and *BMP15* were assessed in cumulus cells **(A)** and oocytes **(B)** supplemented with various concentrations of FGF7 after IVM. The experiment was performed in triplicate. Bars with different letters (^a, b^) indicate significant differences (*p* < 0.05).

The mRNA expression of genes involved in the FGF7 signaling pathway and OSF-related genes in CCs and oocytes after IVM (Figures 6A, B) was evaluated via qPCR. *AKT1* and *PIK3R1* levels were not significantly different from those in the control group (*p* > 0.05). In addition, the expression of c-kit signaling (*c-kit* and *KITLG*) was significantly increased in oocytes treated with 10 ng/mL FGF7 compared to the controls (*p* < 0.05), but no significant alterations in CCs (*p* > 0.05) were observed. In addition, only the *BMP15* OSF-related gene was significantly upregulated in oocytes treated with 10 ng/mL FGF7 (*p* < 0.05), whereas *GDF9* expression was not significantly affected (*p* > 0.05). Notably, the expression levels of *ERK1* and *ERK2* pathway-related genes were slightly different. *ERK1* gene expression levels in CCs were significantly enhanced in the 10 ng/mL and 100 ng/mLFGF7-treated groups relative to those in the control group (*p* < 0.05), and the increase was even higher in the 10 ng/mL-treated group (*p* < 0.05). In oocytes, only the 10 ng/mL FGF7 treatment group exhibited a significant increase in *ERK1* expression (*p* < 0.05). Moreover, significant increases in the *ERK2* gene expression levels were observed in the 100 ng/mL treatment groups for CCs and in the 10 ng/mL treatment group for oocytes (*p* < 0.05, Figures 6A, B).

### 3.6. Effect of FGF7 supplementation in IVM media on developmental potential after PA

**Figure 7:**
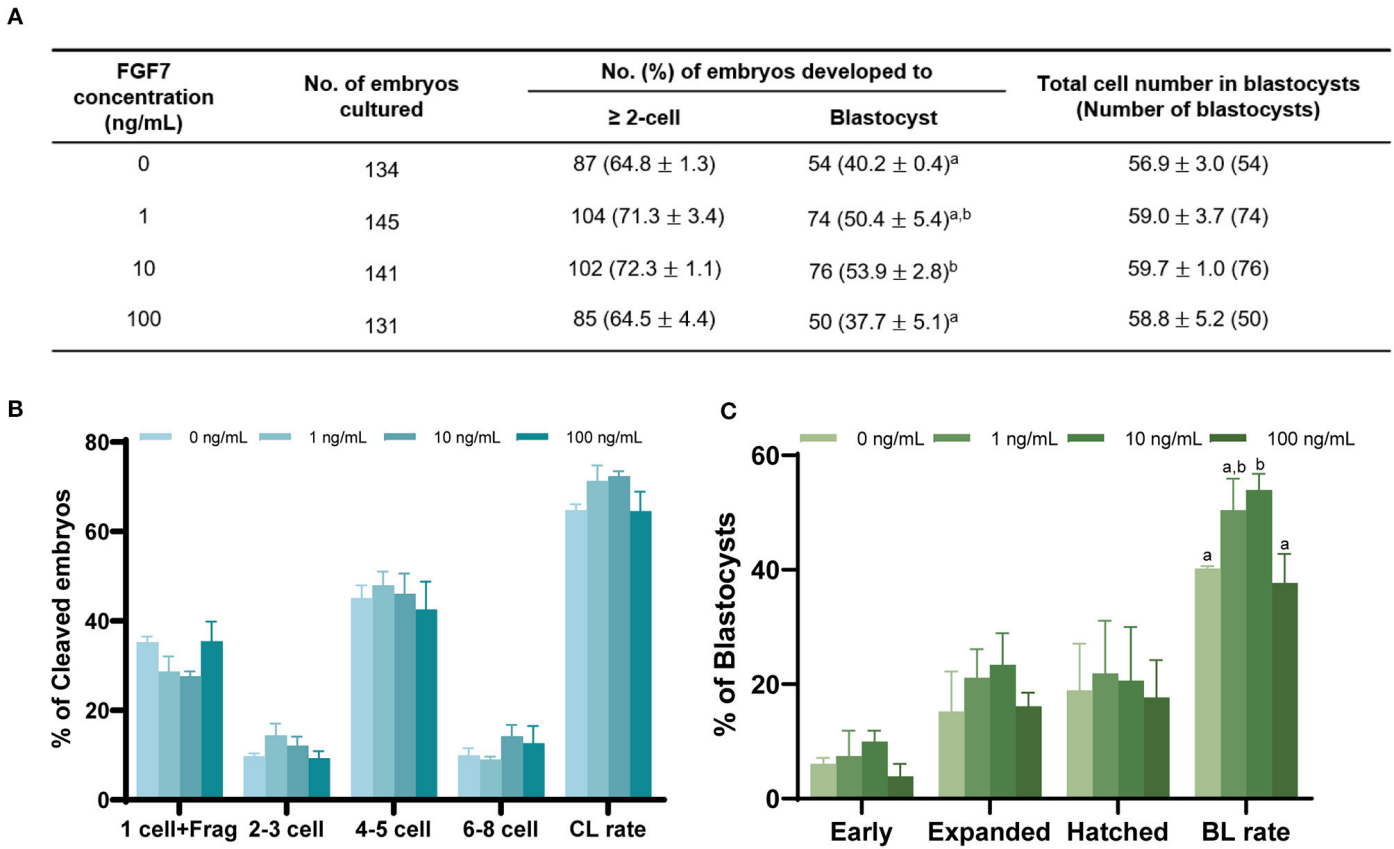
Effect of FGF7 treatment during IVM on embryonic development after PA. **(A)** Effect of FGF7 supplementation during IVM on embryonic development. Effect of different concentrations of FGF7 during IVM on the percentage of PA embryos that developed to the cleavage on day 2 **(B)** the blastocyst stage on day 7. **(C)** Early, early blastocyst; Expanded, expanded blastocyst; Hatched, hatched blastocyst; CL rate, Cleavage rate; BL rate, Blastocyst rate; Frag, Fragmentation. The bars with different letters (^a, b^) are significantly different (*p* < 0.05).

After PA, no significant difference was observed between the control and FGF7-treated groups regarding cleavage rate (*p* > 0.05; Figure 7A). Blastocyst formation rates were significantly higher in the 10 ng/mL FGF7-treated group than in the control group (*p* < 0.05) (Figure 7A). The cleavage patterns of 2–3, 4–5, and 6–8 cells did not differ significantly (*p* > 0.05) (Figure 7B). A comparison of blastocyst formation patterns on day 7 between the groups revealed no significant differences in early, expanded, and hatching blastocyst formation patterns (*p* > 0.05; Figure 7C). The total number of blastocyst cells did not differ substantially from that of the control group (Figure 7A).

## 4. Discussion

Various reproductive technologies have been developed and refined through pig research over the past several decades, from IVM, IVF, and SCNT to animal cloning (31). As a key underlying factor in these procedures, the quality of oocyte maturation *in vitro* substantially impacts fertilization, early embryo survival, gestation maintenance, and fetal development (32, 33).

FGF7 is expressed in endometrial epithelial cells of the porcine uterus (34) and is regulated by progesterone and estradiol (35). Studies on cattle have confirmed the involvement of FGF7 and its receptor in folliculogenesis via angiogenesis stimulation and GCs survival and proliferation, particularly during the final growth of preovulatory follicles (20, 36). Nevertheless, previous FGF7 investigations in porcine ovarian follicles have only detected FGF7 and its receptor at the mRNA level in theca cells and GCs (37). Therefore, to determine whether the FGF7 protein plays a role in porcine follicle development, IHC staining was performed to determine the location of FGF7 and FGFR2IIIb in porcine ovarian follicle cells. FGF7 was expressed in the GCs, CCs, and oocytes of small- and medium-sized follicles. In large follicles, FGF7 was highly expressed in the zona pellucida of oocytes. The localization of FGF7 in this study is slightly differs from the localization in buffalo ovaries reported by Mishra et al. (20), where FGF7 was found only in the theca cells and GCs in the follicles. However, the results of the present study are similar to the localization of FGF7 in sheep follicles, where FGF7 was expressed in oocytes, GCs, theca cells, and stromal cells at all follicular developmental stages (21). This study demonstrated stable FGFR2IIIb expression in GCs, CCs, and the oocyte zona pellucida of small-to-large follicles. However, in buffalo ovaries, FGFR2IIIb mRNA and proteins were significantly expressed only in the GCs of large follicles (20). Although our results were differ than other findings of previous studies on various species, we suggest that FGF7 and its receptor are necessary for follicle development in porcine ovaries.

Oocyte diameter is a parameter used to assess oocyte quality and size, and nuclear maturation is positively correlated in pigs (38). Previous studies in cattle (39, 40) and sheep (41) have demonstrated a distinct relationship between oocyte diameter and developmental capacity. In the present study, compared with untreated control group, the diameter of the FGF7-treated porcine oocytes increased, consistent with the findings of Cho et al. (24) regarding the effects of FGF7 on bovine oocytes. Bovine (24, 42) and mouse (43) studies have shown that FGF7 stimulates oocyte growth *in vitro* by activating the KITLG/c-kit pathway. This study observed a significate increase in KITLG/c-kit pathway-related gene expression in the FGF7-treated oocytes. Although additional steps are required to detect changes in protein levels, in the present study, FGF7 promoted the growth of porcine oocytes *in vitro* by modulating the KITLG/c-kit pathway.

Considering previous studies on the positive correlation between oocyte diameter and oocyte maturation (38), the increase in oocyte diameter following FGF7 treatment may also influence oocyte cytoplasmic and nuclear maturation. Furthermore, the translation of FGF7-encoding mRNA reportedly increased during oocyte maturation from GV to MII, suggesting the involvement of FGF7 in oocyte maturation (44). Therefore, this study evaluated the cytoplasmic and nuclear maturation of oocytes treated with FGF7. Our results revealed that, FGF7 did not affect meiotic progression; however, 10 ng/mL FGF7 promoted the expulsion of the first polar body. Furthermore, oocytes generate ROS during IVM, and the balance between ROS and antioxidants is essential for oocyte development (45). Cytoplasmic GSH is a major cellular redox regulator that protects somatic cells (46) and gametes (47) from oxidative stress. Owing to its vital role in oocyte development, GSH is considered a molecular marker for predicting cytoplasmic maturation (48). In this study, FGF7-treated porcine oocytes exhibited increased GSH levels, indicating improved cytoplasmic maturation and reduced ROS levels. This finding was verified by the results of gene expression, where *Cx43*, an oocyte maturation marker (49–51) and antioxidant factor-related genes *GCLC, NQO1*, and *HMOX1* (52) showed significant elevation in this study.

Oocyte quality can be assessed by detecting apoptosis. Various studies supported the theory that the level of apoptosis is negatively correlated with oocyte quality (53–55). BCL2 family (*BCL2L1*) are negative regulators of cell death that prevent apoptosis induced by numerous stimuli (56, 57), whereas *BAX* is a pro-apoptotic factor (58). The expression levels of the apoptosis regulators *BCL2L1* and *BAX* were examined to determine the quality of the oocytes. FGF7 supplementation during porcine IVM decreased *BAX* expression and increased *BCL2L1* expression. In addition, studies on alveolar epithelial cells, hepatocytes, and keratinocytes have demonstrated the protective effects of FGF7 against apoptosis and oxidative damage (59–61). These results suggest that FGF7 supplementation during IVM improves the cytoplasmic and nuclear maturation of porcine oocytes, thereby enhancing their quality.

FGF7 binds to FGFR2IIIb and trigger the RAS-mitogen-activated protein kinase (MAPK/ERK) signaling pathway (62, 63), which is involved in various functions, such as cell proliferation, differentiation, development, inflammatory response, and apoptosis (64). The increased expression of *ERK1* and *ERK2* in FGF7-treated CCs and oocytes suggests that this pathway plays a role in porcine oocyte maturation. Although further examination is needed, FGF7 regulates the ERK signaling pathway in various cells, such as human endometrial stromal cells (63) and embryonic stem cells (65), to accomplish a wide range of functions.

In the ovaries, the OSF *GDF9* and *BMP15* play critical roles in folliculogenesis, oocyte maturation, and ovulation (66, 67). *GDF9* is essential for regulating oocyte maturation and GCs proliferation and differentiation, whereas *BMP15* has a similar effect because it is a closely related paralogous homolog (68). The existence of a regulatory feedback system between *BMP15* or *GDF9* and granulosa *KITLG* maintains proper expression levels of *BMP15* and *GDF9* in oocytes, which are critical for their physiological functions (69). Considering that there is reciprocal regulation between FGF7 and KITLG/c-kit (42, 43), this study examined changes in *GDF9* and *BMP15* gene expression in FGF7-treated oocytes. FGF7 treatment enhanced *BMP15* expression in porcine oocytes, in agreement with previous results on oocyte maturation. However, no changes in *GDF9* mRNA were observed. Although this finding differs from the previous results for FGF7-treated bovine oocytes (24), in general, FGF7 treatment contributes to the maturation of porcine oocytes *in vitro* by increasing maternal gene expression.

GSH levels in oocytes during IVM are associated with increased developmental competence (70, 71). Studies on oocyte maturation have identified intracellular GSH levels as molecular markers of developmental competence in mature oocytes (72, 73). In particular, for parthenogenesis, a decrease in GSH levels may lead to poor developmental parthenogenesis capacity (74). Although there was no considerable difference in the cleavage rate between FGF7-treated groups after parthenogenic activation in this study, the percentage of cells reaching the blastocyst stage was higher in the FGF7-treated group. This finding is in agreement with the results of previous experiments on GSH. These results suggest that FGF7 supplementation during IVM enhances the developmental capacity of porcine oocytes and increases the number of embryos produced *in vitro*.

Additionally, our study had limitations. The findings of this investigation are primarily confined to *in vitro* studies; therefore, a comparative analysis with *in vivo* developmental processes presents as potential future research. These studies will help to determine whether the effects of FGF7 differ between *in vivo* and *in vitro* conditions. Furthermore, this study is limited to the role of FGF7, and there are many unstudied family members of the FGF family that could be investigated to determine if they can promote oocyte maturation and subsequent development of the oocyte. In addition, this study was limited to porcine species. Future studies could therefore be conducted to compare the differences in the effects of FGF7 on different animal species. Prior empirical evidence suggests an interaction between FGF7 and Placenta-specific protein 1 (Plac1), potentially modulating oocyte meiosis and fertilization via Plac1 (75, 76). Subsequent investigations could direct their focus toward elucidating the role of FGF7 in the IVF outcomes, possibly FGF7 play primary role in IVF-grown blastocysts. Additionally, with regard to late blastocyst development, research in mice demonstrated that FGF7 treatment can promote blastocyst implantation (77). In pigs and humans, FGF7 can regulate the interaction between the developing embryo with the endometrium (63, 78). Therefore, FGF7 may be pivotal in future investigations into the subsequent development of blastocysts.

## 5. Conclusion

This study demonstrates the localization of FGF7 and FGFR2IIIb in porcine ovarian follicles and the positive effects of FGF7 supplementation during IVM on the nuclear and cytoplasmic maturation of porcine oocytes. Furthermore, FGF7 supplementation during IVM enhances the developmental potential of PA-derived porcine embryos. These findings provide new insights into the enhancement of oocyte maturation and subsequent development of porcine oocytes *in vitro*.

## Data availability statement

The original contributions presented in the study are included in the article/Supplementary material, further inquiries can be directed to the corresponding author.

## Ethics statement

Ethical review and approval was not required for the study on animals in accordance with the local legislation and institutional requirements.

## Author contributions

HZ and S-HH: conceptualization, validation, writing—original draft preparation, and writing—review and editing. HZ, HC, DO, MK, LC, SK, and JL: methodology and formal analysis. HZ, HC, DO, MK, LC, JL, AJ, SK, and S-HH: investigation. S-HH: funding acquisition. All authors have read and agreed to the published version of the manuscript.
