# Supplementary material for: Supplementation with fibroblast growth factor 7 during in vitro maturation of porcine cumulus-oocyte complexes improves oocyte maturation and early embryonic development
Source: Front Vet Sci. 2023 Nov 7;10:1250551. doi: 10.3389/fvets.2023.1250551 (PMC10662523; doi:10.3389/fvets.2023.1250551)
Supplement: Supplementary file 1 [file Table_1.DOCX]

Supplementary Material

# Supplementary Table 1

# Primer sequences used for qRT-PCR.

| **mRNA** | **Primer sequences** | **Product size (bp)** | **GenBank**  **accession number** |
| --- | --- | --- | --- |
| *RN18S* | F: 5'-CGCGGTTCTATTTTGTTGGT-3' | 219 | NR_046261.1 |
|  | R: 5'-AGTCGGCATCGTTTATGGTC-3' |  |  |
| *GAPDH* | F: 5’-GTCGGTTGTGGATCTGACCT-3’ | 374 | NC_010447.5 |
|  | R: 5’-TTGACGAAGTGGTCGTTGAG-3’ |  |  |
| *BAX* | F: 5'-TGCCTCAGGATGCATCTACC-3' | 199 | XM_003127290 |
|  | R: 5'-AAGTAGAAAAGCGCGACCAC-3' |  |  |
| *BCL2L1* | F: 5'-AATGACCACCTAGAGCCTTG-3' | 182 | NM_214285 |
|  | R: 5'-GGTCATTTCCGACTGAAGAG-3' |  |  |
| *NRF2* | F: 5’-CCCATTCACAAAAGACAAACATTC-3’ | 72 | XM_021075133.1 |
|  | R: 5’-GCTTTTGCCCTTAGCTCATCTC-3’ |  |  |
| *NQO1* | F: 5’-TATCCTCCTCTGGCCAATTC-3’ | 81 | NM_0011159613.1 |
|  | R: 5’-AGGCGTTTCTTCCACTCTT-3’ |  |  |
| *HMOX1* | F: 5’-AAGGCTTTAAGCTGGTGATG-3’ | 104 | NM_001004027.1 |
|  | R: 5’-GAAGTAGAGGGGCGTGTAG-3’ |  |  |
| *GCLC* | F: 5’-GTTTTGTGAATCAGGACCCTA-3’ | 212 | XM_021098556.1 |
|  | R: 5’-GCTTAGCTGAAGCTTTATTGC-3’ |  |  |
| *PTX3* | F: 5’-AGACTTTATGCCATGGTGCT-3’ | 195 | NM_001244783.1 |
|  | R: 5’-TGACAGTGAGCAATGAACAA-3’ |  |  |
| *CD44* | F: 5’-AGTCAAGAAGGTGAGGCAAA-3’ | 175 | XM_021085286.1 |
|  | R: 5’-TGCCATTGTTAATCACCAGC-3’ |  |  |
| *Has2* | F: 5’ -TTACAATCCTCCTGGGTGGT-3’ | 199 | NM_214053.1 |
|  | R: 5’-TCAAGCACCATGTCGTACT-3’ |  |  |
| *PCNA* | F: 5’-CCTGTGCAAAAGATGGAGTG-3’ | 187 | NM_001291925.1 |
|  | R: 5’-GGAGAGAGTGGAGTGGCTTTT-3’ |  |  |
| *Cx43* | F: 5’-ACTGAGCCCCTCCAAAGAC-3’ | 191 | NM_001244212 |
|  | R: 5’-GCTCGGCACTGTAATTAGC-3’ |  |  |
| *ERK1* | F: 5’-ATCACAGTGGAGGAAGCACT-3’ | 202 | XM_021088019 |
|  | R: 5’-GAGGCATCTGTCCAGGTTAG-3’ |  |  |
| *ERK2* | F: 5’-AGTCCATCGACATCTGGTCT-3’ | 240 | XM_021088019 |
|  | R: 5’-GAGCTTTGGAGTCAGCATTT-3’ |  |  |
| *PI3KR1* | F: 5’-CCACTACCGGAATGAATCTC-3’ | 211 | XM_021076847.1 |
|  | R: 5’-TTCCTGGGAAGTACGGGTAT-3’ |  |  |
| *AKT1* | F: 5’-CCACTACCGGAATGAATCTC-3’ | 208 | NM_001159776.1 |
|  | R: 5’-TTCCTGGGAAGTACGGGTAT-3’ |  |  |
| *c-kit* | F: 5'-GGGAGGATTATCCCAAGTCT-3' | 127 | NM_001044525.1 |
|  | R: 5'-GGGAGGATTATCCCAAGTCT-3' |  |  |
| *KITLG* | F: 5’-CTGTTAGCCATCCCCTACCT-3’ | 195 | XM_021091142.1 |
|  | R: 5’-CACCTCCCATGATCTCTGAC-3’ |  |  |
| *GDF9* | F: 5'-GGTTCCAGCTTCATTCAATC-3' | 120 | NM_001001909.1 |
|  | R: 5'-ACAATCCAGTTGTCCCACTT-3' |  |  |
| *BMP15* | F: 5'-CCATCATCCAGAACCTTGTC-3' | 154 | NM_001005155.2 |
|  | R: 5'-CAGGACTGGGCAATCATATC-3' |  |  |

F: Forward, R: Reverse
